# T cell responses and clinical symptoms among infants with congenital cytomegalovirus infection

**DOI:** 10.1172/jci.insight.171029

**Published:** 2024-09-24

**Authors:** Alexandra K. Medoro, Ravi Dhital, Pablo J. Sánchez, Kaitlyn Flint, Brianna Graber, Traci Pifer, Rachelle Crisan, William C. Ray, Christopher C. Phelps, Jonathan R. Honegger, Jing Peng, Ursula Findlen, Prashant Malhotra, Oliver Adunka, Masako Shimamura

**Affiliations:** 1 Department of Pediatrics, Division of Infectious Diseases, and; 2 Department of Pediatrics, Division of Neonatology, Nationwide Children’s Hospital and The Ohio State University College of Medicine, Columbus, Ohio, USA.; 3 Center for Vaccines and Immunity and; 4 Center for Perinatal Research, Abigail Wexner Research Institute at Nationwide Children’s Hospital, Columbus, Ohio, USA.; 5 Interdisciplinary Graduate Program in Biophysics, The Ohio State University, Columbus Ohio, USA.; 6 IT Research and Innovations Group, Nationwide Children’s Hospital, Columbus, Ohio, USA.; 7 Center for Clinical and Translational Science, Abigail Wexner Research Institute at Nationwide Children’s Hospital, Columbus, Ohio, USA.; 8 Center for Biostatistics, The Ohio State University, Columbus, Ohio, USA.; 9 Department of Audiology and; 10 Department of Otolaryngology, Nationwide Children’s Hospital and The Ohio State University College of Medicine, Columbus, Ohio, USA.

**Keywords:** Immunology, Infectious disease, Neurodevelopment, T cells

## Abstract

**BACKGROUND:**

Congenital cytomegalovirus (cCMV) infection can cause developmental impairment and sensorineural hearing loss (SNHL). To determine the relationship between immune responses to cCMV infection and neurologic sequelae, T cell responses were compared for their connection to clinical symptoms at birth and neurodevelopmental outcomes.

**METHODS:**

Thirty cCMV-infected and 15 uninfected infants were enrolled in a single-center prospective observational case-control study. T cell pp65-specific cytokine responses; CD57, CD28, and PD-1 expression; and memory subsets were compared.

**RESULTS:**

Infected neonates (73% symptomatic at birth) lacked pp65-specific cytokine-secreting T cells, with elevated frequencies of CD57^+^, CD28^–^, and PD-1^+^CD8^+^ T cells and effector memory subsets. Though frequencies overlapped between cCMV symptom groups, asymptomatic infants had higher frequencies of CD57^+^PD-1^+^CD8^+^ T cells. Neonates with subsequent developmental delay lacked detectable CMV-specific T cell responses, with patterns resembling those of uninfected infants. Two children with progressive SNHL had high frequencies of PD-1^+^CD8^+^ T cells over the first year compared with children without progressive SNHL.

**CONCLUSION:**

Similar to published reports, neonatal viral antigen–specific cytokine-secreting T cell responses were not detected, but overall patterns indicate that globally differentiated memory CD8^+^ T cell populations were induced by cCMV infection, with higher frequencies of terminally differentiated PD-1^+^CD8^+^ T cells potentially associated with asymptomatic infection. In this cohort, a lack of in utero T cell differentiation was associated with developmental delay, and high frequencies of PD-1^+^CD8^+^ T cells persisted only in children with progressive SNHL. Further work is needed to define the specificity of these T cells and their mechanistic connection to these outcomes.

**FUNDING:**

This study was funded through an intramural research award at Nationwide Children’s Hospital, the Pediatric Infectious Disease Society Fellowship Award funded by Stanley and Susan Plotkin and Sanofi Pasteur, the Abigail Wexner Research Institute at Nationwide Children’s Hospital, and the Pichichero Family Foundation Vaccines for Children Initiative Research Award from the Pediatric Infectious Diseases Society Foundation.

## Abstract 2

T cell responses of infants with congenital cytomegalovirus infection vary. Lack of T cell differentiation and persistence of PD-1 expression were associated with disease sequelae.

## Introduction

Congenital cytomegalovirus (cCMV) infection is the leading nongenetic cause of childhood sensorineural hearing loss (SNHL), and it can cause permanent neurodevelopmental impairments (1–3). The majority of children with cCMV infection have clinically inapparent or “asymptomatic” infection at birth (85%–90%), while the minority (10%–15%) have clinically apparent or “symptomatic” disease at birth (4). Clinical findings at birth can include symptoms, such as microcephaly, hepatosplenomegaly, and intrauterine growth restriction, which is also known as growth small for gestational age; lab abnormalities, such as direct hyperbilirubinemia, thrombocytopenia, or transaminitis; ophthalmologic manifestations, such as chorioretinitis; or CNS imaging findings such as intracranial calcifications or errors of neuronal migration. Neurologic sequelae of cCMV infection include cerebral palsy, intellectual impairment, vision loss, and SNHL (1, 5). Intellectual impairment is associated with microcephaly and chorioretinitis at birth, but children without these findings may also have abnormal neurodevelopmental outcomes, including those who are asymptomatic at birth (6, 7). Early-onset SNHL (eoSNHL) due to cCMV is present at birth and may be detected by newborn hearing screening, whereas late-onset SNHL (loSNHL) develops later in childhood and may manifest as language delay. SNHL can be unilateral or bilateral and may be progressive and/or fluctuating over time (5, 8). While infants with symptomatic infection have a higher risk of SNHL (27%–50%), specific clinical symptoms at birth do not predict risk for progressive hearing loss (3, 8, 9). In addition, 10%–12% of infants with asymptomatic infection will also develop loSNHL (3, 8, 10). Given the higher total number of children born with asymptomatic cCMV infection, the majority of cCMV-related SNHL has been estimated to occur in asymptomatic infants (10). Together, these data demonstrate that clinical parameters do not reliably predict cCMV-induced neurologic impairment. Antiviral therapy with 6 months of ganciclovir or valganciclovir has been shown to mitigate progression of SNHL and may have a positive effect on neurodevelopment; however, it is only currently recommended for moderately to severely symptomatic infants and may induce dose-limiting neutropenia (11–13). Identification of biomarkers for risk of neurodevelopmental or hearing impairment in infants with asymptomatic or mild cCMV infection would enable targeted antiviral therapy for those infants most likely to benefit from treatment while avoiding drug exposure for infants at low risk for adverse neurologic outcomes.

Blood transcriptional profiling using microarray technology has shown upregulated expression of genes related to T cells in infants with cCMV infection compared with uninfected infants, but the transcriptional signatures of infants with symptomatic (*n* = 49) and asymptomatic (*n* = 31) cCMV infection were indistinguishable from one another (14). CMV antigen-specific CD8^+^ T cells are detectable in cord blood of infants with cCMV infection, along with global oligoclonally expanded populations that express markers of differentiation and activation (15–17). These global CD8^+^ T cell responses can also be detected in fetal blood as early as 22 weeks gestational age (18, 19). Together, these studies demonstrate the capacity of fetal CD8^+^ T cells to differentiate and expand in response to CMV antigens in utero. However, these cells have poor antigen-specific cytokine-secreting and proliferative capacity, lack polyfunctionality, and express markers of T cell exhaustion such as PD-1 (15, 16, 20–26). A large historical study (*n* = 104, 33 symptomatic and 71 asymptomatic neonates) demonstrated that proliferative responses to CMV antigens remain poorly detected for up to 5–7 years after birth (27). However, recent studies utilizing contemporary methodologies showed that CMV antigen-specific cytokine responses (Quantiferon-CMV, Qiagen) were detected among 16 of 20 (80%) asymptomatic neonates but none of 10 (0%) symptomatically infected neonates and that T cell responses (via ELISpot, MilliporeSigma) broadened and gained avidity over 1–3 years among 4 cCMV-infected infants (2 symptomatic, 2 asymptomatic) (28, 29). These global and antigen-specific T cell responses to cCMV infection have not been analyzed in context of clinical symptoms at birth and neurologic sequelae. Therefore, the objective of this study was to compare the T cell responses at birth and longitudinally among cCMV-infected infants for their connection to clinical manifestations at birth and neurodevelopmental outcomes.

## Results

### Characteristics of the study population.

Thirty infants with cCMV infection and 15 cross-sectionally age-matched, CMV-uninfected controls were enrolled in the study (Supplemental Table 1; supplemental material available online with this article; https://doi.org/10.1172/jci.insight.171029DS1). The demographic and clinical characteristics of these participants are shown in Table 1. The infants with cCMV were not significantly different from the uninfected infants with respect to sex distribution, gestational age, birth weight, or head circumference; however, those with cCMV infection had significantly shorter birth lengths, with other birth indices also trending toward smaller size. The majority (73%) of infants with cCMV infection were symptomatic at birth, with 6% displaying isolated eoSNHL (eoSNHL); 40% with symptomatic cCMV not involving the CNS (Sx No CNS); and 27% with symptomatic cCMV involving the CNS (Sx +CNS). The majority (70%) received antiviral therapy with ganciclovir and/or valganciclovir. Of the 30 infants with cCMV infection, 14 infants (47%) had any neurodevelopmental impairment, defined as developmental delay (DD) and/or SNHL, with 9 (30%) having DD and 9 (30%) with SNHL. One infant in the cohort had only 2 months of follow-up so was not included in the outcome analysis.

### CMV pp65-specific cytokine responses at ≤60 days of age.

**Figure 1:**
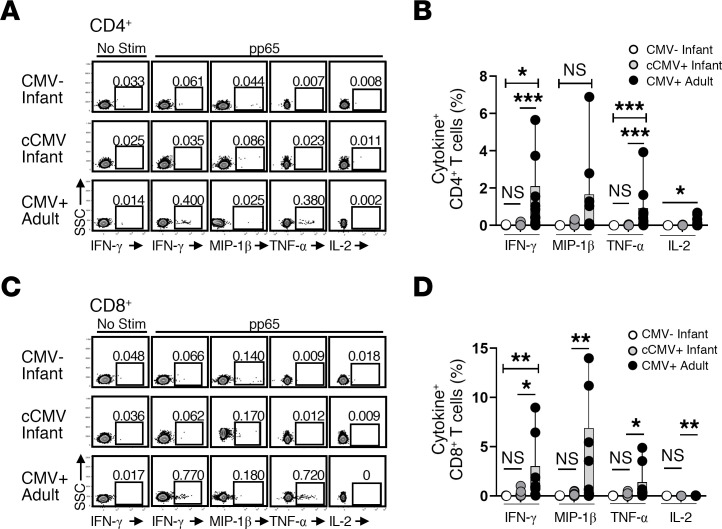
CMV pp65-specific T cell responses in cCMV-infected neonates. PBMCs from cCMV-infected infants (*n* = 21) or uninfected infants (*n* = 5) at ≤60 days of age and CMV-seropositive adults (*n* = 10) were unstimulated or stimulated with CMV pp65 peptide pool and analyzed by intracellular cytokine staining and flow cytometry. Representative flow plots (**A** and **C**) and aggregated data (**B** and **D**) are shown for frequencies of CD4^+^ (**A** and **B**) and CD8^+^ (**C** and **D**) T cells expressing IFN-γ, MIP-1β, TNF-α, and IL-2 after pp65 stimulation (background subtracted from unstimulated frequencies) for CMV-uninfected infants, cCMV-infected infants, and CMV-seropositive (CMV+) adults. Groups were compared using the Kruskal-Wallis test (**B** and **D**) with Dunn’s multiple comparisons. *P < 0.05; **P < 0.01; ***P < 0.001; ns, not significant (*P* > 0.05).

The frequencies of pp65-specific T cells expressing Th1/Tc1-biased cytokines (IFN-γ, MIP-1β, TNF-α, and IL-2) were compared between 21 cCMV-infected and 5 uninfected neonates with blood samples available at ≤60 days of age and with 10 CMV-seropositive adults (Figure 1). Representative flow plots show cytokine expression of CD4^+^ (Figure 1A) and CD8^+^ T cells (Figure 1C) with and without pp65 stimulation for an uninfected infant, an infant with cCMV infection, and a CMV-seropositive adult. For each group, frequencies of CMV pp65-specific CD4^+^ (Figure 1B) and CD8^+^ (Figure 1D) T cells expressing IFN-γ, MIP-1β, TNF-α, and IL-2 are shown. Frequencies of CMV pp65-specific CD4^+^ and CD8^+^ T cells expressing IFN-γ, MIP-1β, TNF-α, and IL-2 were similar between infants with and without cCMV infection. CMV-seropositive adult participants had significantly higher frequencies of pp65-specific CD4^+^ and CD8^+^ T cells expressing IFN-γ, TNF-α, and MIP-1β compared with uninfected participants. Of the 21 cCMV-infected infants, 15 (71%) had complete blood counts performed, and these infants had a median absolute lymphocyte count (ALC) of 6,600 cells/μL blood (range, 4,300–9,900 cells/μL blood). These ALCs are within the published range of 4,000–12,600 cells/μL blood for healthy infants at 1–6 weeks of age and are higher of those of healthy adults (range, 1,100–2,400 cells/μL) (30). These data indicate that cCMV-infected infants lack CMV pp65-specific cytokine-secreting CD4^+^ and CD8^+^ T cell responses in the neonatal period, even with lymphocyte counts similar to uninfected infants and higher than seropositive adults. To assess global cytokine-secreting capacity, cells were stimulated with Staphylococcal enterotoxin B (SEB), and frequencies of cytokine-secreting cells were quantified for each group (Supplemental Figure 1) The cytokine responses were similar between uninfected and cCMV-infected infants, whereas adults had significantly higher IFN-γ, MIP-1β, TNF-α, and IL-2 secretion in response to SEB. These results are consistent with other reports showing lower cytokine-secreting T cell responses to SEB in newborns compared with adults (31, 32), which may account for the lower CMV antigen-specific responses observed in the cCMV-infected neonates.

### T cell markers of differentiation and memory at ≤60 days of age.

**Figure 2:**
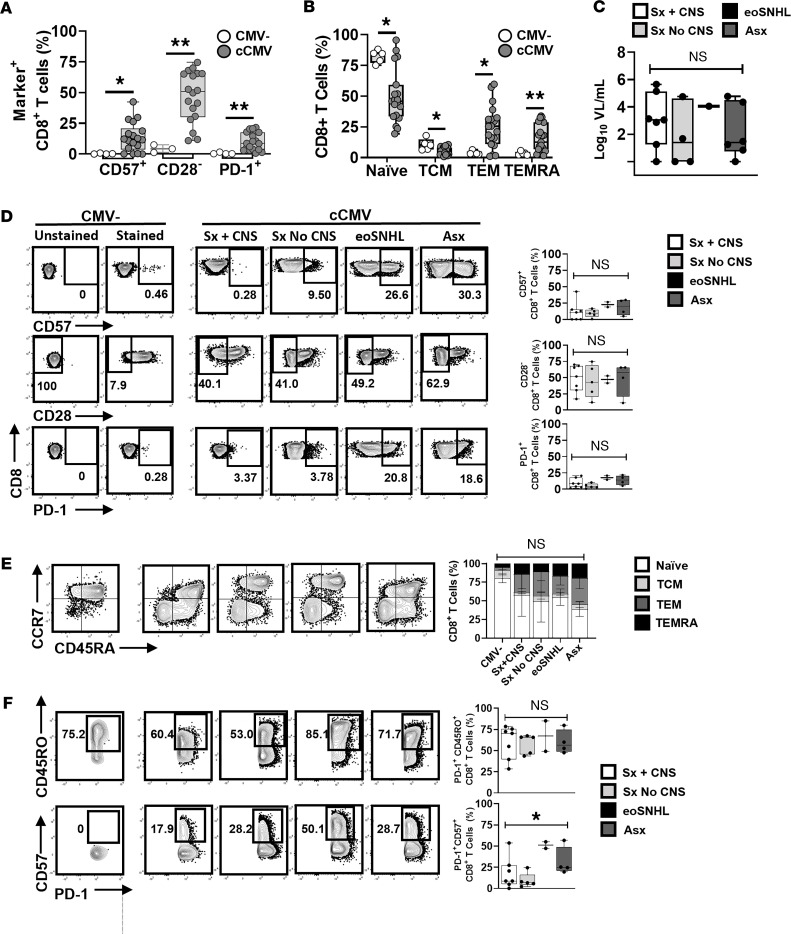
Global CD8^+^ T cell differentiation and memory in cCMV-infected infants categorized by clinical symptoms at birth. PBMCs of cCMV-infected (*n* = 18) or uninfected infants (*n* = 4) at ≤60 days of age were analyzed by flow cytometry for frequencies of global populations of CD8^+^ T cells expressing CD57, CD28, PD-1, CD45RO, CCR7, and CD45RA. Uninfected and cCMV-infected groups (**A** and **B**) and cCMV-infected individuals grouped by clinical symptoms at birth (**C**–**F**) were compared. Sx + CNS, symptomatic with CNS findings (*n* = 7); Sx no CNS, symptomatic without CNS findings (*n* = 5); eoSNHL, early onset sensorineural hearing loss (*n* = 2); Asx, asymptomatic (*n* = 6). (**A**) Frequencies of CD57^+^, CD28^–^, or PD-1^+^CD8^+^ T cells between infants with cCMV infection (*n* = 18) and uninfected infants (*n* = 4). (**B**) Frequencies of naive (CCR7^+^/CD45RA^+^), central memory (Tcm; CCR7^+^/CD45RA^–^), effector memory (Tem; CCR7^–^/CD45RA^–^), and effector memory expressing RA (Temra; CCR7^–^/CD45RA^+^) subsets for infants with cCMV (*n* = 18) and uninfected infants (*n* = 5). (**C**) Serum viral loads of infants according to clinical symptom group. (**D**) Representative flow plots of CD8^+^ T cells stained for CD57, CD28, and PD-1 for an uninfected infant (stained and unstained to show gating) and then for cCMV-infected infants (stained only) from each clinical symptom group, with aggregated data shown in graphs. (**E**) Representative flow plots of memory markers CCR7/CD45RA shown for an uninfected infant and cCMV-infected infants from each clinical symptom group, with graph showing proportions of memory subsets by group. (**F**) Representative flow plots and graphs showing frequencies of PD-1^+^CD8^+^ T cells coexpressing either CD45RO or CD57. Comparisons were made using the Mann-Whitney *U* test (**A**, **B**, and **D**), Kruskal-Wallis test (**C** and **F**) with Dunn’s multiple comparisons, or 2-way ANOVA (**E**). *P < 0.05; **P < 0.01; ns, not significant (*P* > 0.05).

Next, global CD8^+^ T cell populations (Figure 2) from cCMV-infected and uninfected infants at ≤60 days of age were assessed for expression of markers of terminal differentiation (CD57^+^, CD28^–^), inhibition (PD-1^+^), and memory (CCR7, CD45RA, CD45RO). Of the neonatal cohort, 18 cCMV-infected infants and 5 uninfected infants had sufficient PBMC quantities for this analysis. Infants with cCMV infection had higher frequencies of CD57^+^, CD28^–^, and PD-1^+^CD8^+^ T cells compared with those of uninfected infants (Figure 2A). Comparing the CD8^+^ T cell memory subset distribution, the cCMV-infected infants had a lower proportion of naive CD8^+^ T cells (CCR7^+^CD45RA^+^) than uninfected infants, with a higher proportion of effector memory ([Tem] CCR7^–^CD45RA^–^) and effector memory expressing RA ([Temra] CCR7^–^CD45RA^+^) subsets (Figure 2B). The T cells of cCMV-infected infants were next compared according to clinical symptoms (Sx + CNS, *n* = 7; Sx No CNS, *n* = 5; eoSNHL, *n* = 2; Asx, *n* = 6). Blood viral loads (Figure 2C); frequencies of CD57^+^, CD28^–^, and PD-1^+^CD8^+^ T cells (Figure 2D); and memory subset distribution (Figure 2E) were similar among the clinical symptom groups. However, asymptomatic infants and those with isolated eoSNHL had higher frequencies of PD-1^+^CD8^+^ T cells coexpressing CD57 (Figure 2F). Absolute numbers of cells/μL blood were calculated for individuals in each group (Supplemental Figure 2). ALCs were available for 100% (*n* = 7 of 7) of the Sx + CNS, 60% (*n* = 3 of 5) of the Sx No CNS, 50% (*n* = 1 of 2) of the eoSNHL, and 67% (*n* = 4 of 6) of the Asx group. Results were similar to frequency comparisons, with a significant difference found only for CD57^+^PD-1^+^CD8^+^ T cells/μL blood (Supplemental Figure 2C). Together, these data indicate that globally differentiated memory CD8^+^ T cell populations are induced by cCMV infection and that a lack of systemic cCMV symptoms is associated with higher frequencies of terminally differentiated CD8^+^ T cells expressing PD-1.

In contrast, CD4^+^ T cells in cCMV infection only had higher CD28^–^ frequencies, with no apparent differences in CD57^+^ or PD-1^+^ frequencies compared with uninfected infants (Supplemental Figure 3A). There was no difference in the distribution of naive, central memory (Tcm), Tem, or TEMRA subsets for CD4^+^ cells between infants with or without cCMV infection (Supplemental Figure 3B). Together, these data indicate that cCMV infection promotes differentiation of a CD28^–^CD4^+^ T cell population but is not associated with a global terminally differentiated memory CD4^+^ T cell phenotype as seen for the CD8^+^ T cells.

### T cell responses in infants with cCMV and neurodevelopmental impairment.

**Figure 3:**
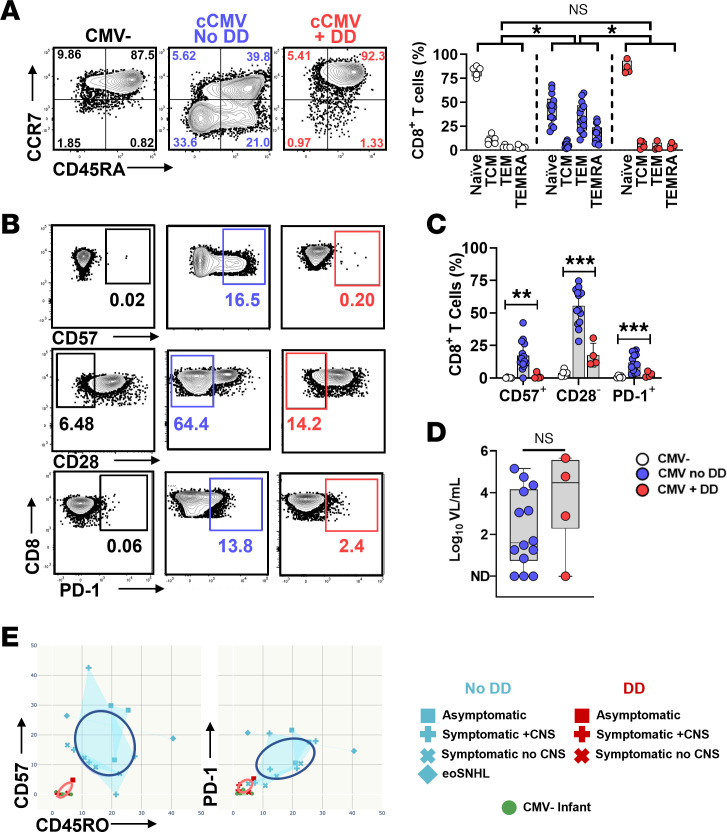
Association of T cell phenotypes with developmental delay. The neonatal T cell phenotypes (≤60 days of age) of cCMV-infected infants with developmental delay (DD, *n* = 4) were compared with those with normal development (No DD, *n* = 14) and uninfected controls (*n* = 5). (**A**) CD8^+^ T cell memory subset distribution and (**B** and **C**) frequencies of CD57^+^, CD28^–^, or PD-1^+^CD8^+^ T cells were compared between groups. (**D**) Plasma viral loads were compared between infants with and without DD. (**E**) Correlation plots show frequencies of CD45RO^+^ and either CD57^+^ or PD-1^+^CD8^+^ T cells by clinical symptom group (shown as shapes connected by lines) and outcomes (red, DD; blue, no DD). Ellipses show 95% CIs for DD/no DD groups. Comparisons were made using Mann-Whitney *U* tests (**D**), Kruskal-Wallis tests with Dunn’s comparisons (**A** and **C**), and SD ellipses (**E**) as appropriate. *P < 0.05; **P < 0.01; ns, not significant (*P* > 0.05).

For the 18 cCMV-infected infants with T cell data at ≤60 days of age and at least 12 months of clinical follow-up, 4 infants had DD and 14 had normal development (No DD) (Figure 3). Infants with DD had higher proportions of naive T cells and lower proportions of Tem and Temra cells at ≤60 days of age than those with normal development, and their memory distribution was statistically similar to that of uninfected infants of similar age (Figure 3A). In addition, infants without DD had higher frequencies of CD57^+^, CD28^–^, and PD-1^+^CD8^+^ T cells than infants with DD and uninfected controls (Figure 3, B and C). Absolute lymphocyte counts were similar between groups (No DD, *n* = 10; DD, *n* = 4) (Supplemental Figure 4, A–C). Absolute cell counts of CD57^+^, CD28^–^, and PD1^+^CD8^+^ T cells/μL blood (Supplemental Figure 4B) were significantly higher in the no DD group compared with the DD group, corroborating the frequencies shown in Figure 3C. As CNS findings at birth correlate with neurodevelopmental impairment (6, 7), we also analyzed only the subgroup of children with CNS findings at birth and confirmed that early CD8^+^ T cell differentiation still distinguished those with and without eventual DD (Supplemental Figure 4D). However, blood viral loads at ≤60 days of age were similar between groups with and without DD (Figure 3D). Correlation plots of CD45RO^+^, CD57^+^, and PD-1^+^CD8^+^ T cells were analyzed for clinical symptoms at birth (as represented with symbols in Figure 3E) and neurodevelopmental outcome (blue, no DD; red, DD; Figure 3E) and showed that groups clustered according to outcomes but not clinical symptoms at birth. Notably, 1 asymptomatic infant developed DD (red square; Figure 3E), and this infant’s neonatal T cell responses clustered with those of other infants with DD but not with the asymptomatic infants with normal developmental outcome (blue squares; Figure 3E). However, longitudinal analysis showed that the frequencies of CD57^+^ and PD-1^+^CD8^+^ T cells became similar between groups over the first year of age (Supplemental Figure 5).

**Figure 4:**
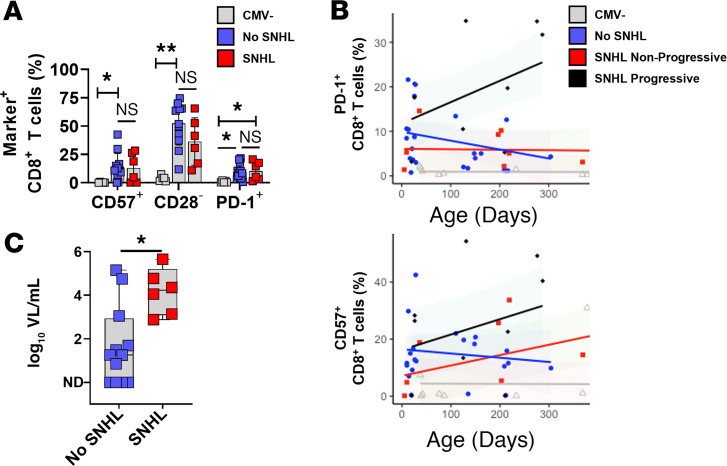
Association of T cell phenotypes with SNHL. The cCMV-infected infants with SNHL (*n* = 9) were compared with those with normal hearing (*n* = 14) and to uninfected controls (*n* = 5). (**A**) For infants with samples available at ≤60 days of age, frequencies of CD57^+^, CD28^–^, or PD-1^+^CD8^+^ T cells were compared among those with (*n* = 6) and without (*n* = 12) SNHL and among uninfected controls (*n* = 5). (**B**) For all infants with longitudinal samples available for analysis, frequencies of PD-1^+^ and CD57^+^ CD8^+^ T cells are shown over the first 365 days of age for those with no SNHL (*n* = 14), nonprogressive SNHL (*n* = 6), and progressive SNHL (*n* = 3), with 95% CIs shown as shaded areas. (**C**) Plasma viral loads at ≤60 days of age were compared between cCMV-infected groups with and without SNHL. Comparisons were made using Mann-Whitney *U* tests (**C**), Kruskal-Wallis tests with Dunn’s comparisons (**A**), and linear-mixed models (**B**). *P < 0.05; **P < 0.01; ns, not significant (*P* > 0.05).

We next assessed cCMV groups with and without SNHL (Figure 4). Frequencies of CD57^+^, CD28^–^, and PD-1^+^CD8^+^ T cells were similar at ≤60 days of age between cCMV-infected infants with (*n* = 9) and without (*n* = 14) SNHL (Figure 4A). However, 2 of 3 infants with progressive SNHL over the first year of age had persistently elevated frequencies of PD-1^+^CD8^+^ T cells compared with infants with nonprogressive SNHL (*n* = 6), those with normal hearing (*n* = 14), and uninfected controls (*n* = 5), while frequencies of CD57^+^CD8^+^ T cells did not differ longitudinally between these groups (Figure 4B). Absolute lymphocyte counts (Supplemental Figure 6, A and B) were compared over time between groups with normal hearing (*n* = 12 of 14, 86%), nonprogressive SNHL (*n* = 5 of 6, 83%), and progressive SNHL (*n* = 3 of 3, 100%), as well as absolute PD-1^+^CD8^+^ T cells/μL blood (Supplemental Figure 6C), with colored lines showing participants with repeated observations over time. These data confirmed that the observation that 2 of the 3 infants with progressive SNHL had persistently higher absolute quantities of PD-1^+^CD8^+^ T cells over time compared with those with nonprogressive or no SNHL. Serum viral loads were significantly higher in the group with SNHL at birth, compared with those with normal hearing (Figure 4C). Together, these data suggest that while these immune phenotypes do not vary based on clinical presentation at birth, temporal persistence of PD-1^+^CD8^+^ T cells may be observed in children with progressive SNHL.

### Longitudinal CMV-specific T cell responses.

Frequencies of pp65-specific cytokine-expressing CD4^+^ and CD8^+^ T cells were evaluated at approximately 3-month intervals over the first year of age and remained similar to those observed during the neonatal period for both cCMV-infected (*n* = 30) and uninfected (*n* = 15) infants (Supplemental Figure 7), indicating that antigen-specific T cell responses remain low over the first year of age among cCMV-infected infants.

**Figure 5:**
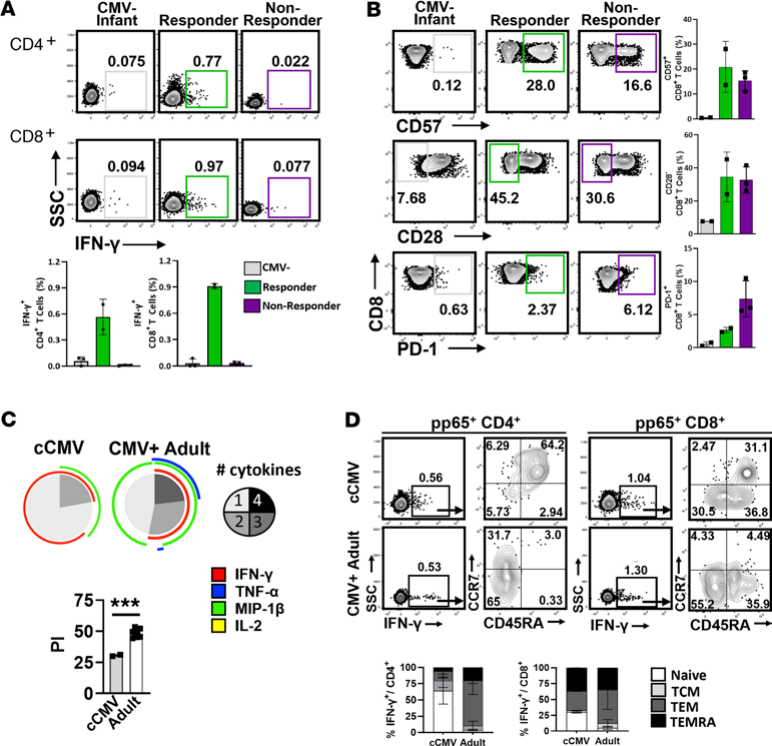
CMV antigen-specific T cell responses during the second year of age. PBMCs from cCMV-infected infants (*n* = 5) and uninfected controls (*n* = 3) at 12–18 months age were analyzed by flow cytometry in comparison with CMV-seropositive adults (*n* = 10). (**A**) CMV pp65-specific CD4^+^ and CD8^+^ T cell responses are shown for uninfected (*n* = 3), cCMV IFN-γ^+^ responder (*n* = 2), and cCMV IFN-γ^–^ nonresponder (*n* = 3) infants as representative flow plots and graphs. (**B**) Representative flow plots and graphs of CD57^+^, CD28^–^, or PD-1^+^CD8^+^ T cells are shown for uninfected children (*n* = 2; 1 infant had insufficient PBMCs for staining) and for cCMV responder and nonresponder infants. (**C**) CD8^+^ T cell polyfunctionality of cCMV responders was compared with that of CMV-seropositive adults using SPICE software and the polyfunctionality index (PI). (**D**) CCR7/CD45RA memory subset distribution of CMV pp65^+^IFN-γ^+^ CD4^+^ and CD8^+^ T cells is shown for cCMV-infected infants and CMV-seropositive adults as representative flow plots and graphs. Comparison were made using Mann Whitney *U* test (**C**). ***P < 0.001.

However, during the second year of age, 5 cCMV-infected children and 3 age-matched uninfected children had PBMCs available for analysis. Of these cCMV-infected children, 2 infants had detectable pp65-specific cytokine-secreting T cells (“responders”), whereas 3 others did not (“nonresponders”) (Figure 5A). While small sample size prohibited formal statistical analysis of this subgroup, there was no difference in frequencies of CD57^+^ or CD28^–^CD8^+^ T cells between nonresponders and responders; however, responders had low frequencies of PD-1^+^CD8^+^ T cells compared with the nonresponders and appeared more similar to frequencies observed in participants acting as healthy controls (Figure 5B). Comparing the pp65-specific cytokine responses of responders to those of CMV-seropositive adults, the polyfunctionality index (PI) of the 2 cCMV responder infants was lower than the PI of CMV-seropositive adults (*n* = 10) (Figure 5C). Of the CMV pp65-specific IFN-γ^+^ CD4^+^ and CD8^+^ T cells, the responder infants had higher frequencies of naive (CCR7^+^ CD45RA^+^) CD4^+^ T cells than seropositive adults and fewer Tem T cells as well as a higher proportion of naive CD8^+^ T cells (Figure 5D). Together, these data suggest that CMV-specific T cell responses are detectable in some but not all cCMV-infected infants during the second year of age, may be associated with low frequencies of PD-1^+^CD8^+^ T cells, and have characteristics of primary responses with low polyfunctionality.

## Discussion

In this prospective observational case-control study, terminally differentiated CD8^+^ T cells lacking pp65-specific cytokine secretion and expressing the inhibitory receptor, PD-1, were observed after cCMV infection, consistent with published reports (15, 16, 22, 33, 34). Similar to other studies, the CMV antigen-specific T cell responses remained low through the first year of age and became detectable in some but not all children during the second postnatal year, with cytokine-secreting responses associated with low frequencies of PD-1^+^CD8^+^ T cells. These later data support the premise put forth by Huygens et al. (16) that cCMV infection induces CD8^+^ T cell exhaustion. In that study, T cells from cord blood PBMCs of cCMV-infected infants were found to express PD-1 and had poor antigen-specific proliferative and cytokine-secreting responses, which increased after in vitro treatment with anti-PDL1/2 antibodies, consistent with reversible (“progenitor”) T cell exhaustion (33, 34). Our study further examined PD-1 expression longitudinally and showed that PD-1 expression diminishes upon detection of CMV-specific IFN-γ^+^ Th1 cells in the second year of age. These findings suggest that CD8^+^ T cells undergo antigen-specific differentiation in utero but may acquire an exhausted phenotype, possibly due to continuous viral antigen exposure and a lack of CD4^+^ T cell help, which may resolve with the development of antiviral cytokine-secreting Th1 cells.

Similar to other published reports (15, 16, 22), the infants in our cohort expressed globally elevated frequencies of terminally differentiated and memory CD8^+^ T cell frequencies. However, these frequencies varied broadly across the cohort and, in extreme cases, appeared phenotypically similar to the T cell profiles of uninfected neonates. This variation in CD8^+^ T cell differentiation has not previously been described and might be attributable to the difference between our cohort and those report by other groups, in that some reported findings among groups predominantly affected by asymptomatic infection (70%–77%) compared with ours (27%) or reported data from infants >60 days of age. In our study, the range of neonatal T cell responses overlapped among clinical symptom groups, consistent with a blood transcriptomic study that showed no significant differences in the T cell transcriptional signatures of infants with symptomatic and asymptomatic infection (14).

Our study shows that the neonatal T cell phenotypes induced by cCMV infection differed according to neurodevelopmental outcome. Infants with later DD had high frequencies of undifferentiated and naive T cells in the neonatal period, resembling those of uninfected neonates, whereas those with normal developmental outcomes had high frequencies of the differentiated memory CD8^+^ T cells that have been reported in other studies (16, 22). Importantly, 1 infant with asymptomatic infection and later DD had a neonatal CD8^+^ T cell phenotype clustering with the DD group but not with that of other asymptomatically infected children with normal developmental outcomes. Our data therefore suggest that a lack of in utero CD8^+^ T cell responses to cCMV infection may be associated with later DD, irrespective of clinical symptoms at birth, a finding that warrants further study among a larger cohort of cCMV-infected children.

Clinical literature indicates that DD and SNHL are independent outcomes after cCMV infection (3, 6, 7, 9). Consistent with these studies, we found that the neonatal T cell responses did not differ between infants with and without SNHL, in contrast to the differences observed between the groups with and without DD. In this small cohort, 2 of the 3 children with progressive SNHL had persistently elevated frequencies of PD-1^+^CD8^+^ T cells over the first year of age compared with infants with nonprogressive SNHL and normal hearing. While limited by a small sample size, this observation has not previously been described to our knowledge and supports the need for larger studies to identify the exact mechanisms by which cCMV-induced T cells may permit or inhibit progressive SNHL, which is an important late outcome of cCMV particularly among infants who are asymptomatically infected at birth.

In our study, some cCMV-infected neonates appeared to lack differentiated memory T cell responses to congenital infection, and their T cells phenotypically resembled the naive T cells of CMV-uninfected children. In murine models, congenital infection with lymphocytic choriomeningitis virus (LCMV) induces immune ignorance, wherein virus-infected pups lack detectable LCMV-specific T cell responses, presumably due to in utero deletion of antigen-specific clones (35). Postnatally, these pups had persistent viral replication that was controlled by adoptive transfer of T cells from LCMV-immune adults, after which the pups became capable of generating endogenous thymus-dependent LCMV-specific T cells (36, 37). These studies indicate that a congenital viral infection in mice can induce T cell ignorance to viral antigens, which can be reversed postnatally by interrupting viral antigen exposure. In contrast, murine models using murine CMV (MCMV) have not demonstrated this immune ignorance. MCMV does not cross the murine placenta, so various models have been developed to analyze CNS infection by inoculating MCMV in utero or via intracerebral or intraperitoneal injection immediately after birth (38–44). In these models, MCMV induces a robust T cell response rather than immunologic ignorance. It is possible that the highly disparate gestational timing and routes of infection in the LCMV and MCMV models could contribute to these observed differences in T cell responses. Our results in clinical cCMV infection suggest that cCMV infection may induce a spectrum of responses overlapping those observed in the LCMV or MCMV models. As the infection route is universally transplacental in human cCMV infection, these differences could perhaps be related to gestational timing of infection. Although the exact pathogenesis underlying our observations in the cCMV-infected newborns is unknown, it is notable that this apparent lack of T cell response to in utero infection may be associated with risk for later DD.

This study has several limitations. Study participants predominantly had symptomatic infection, which might bias the immunologic analyses toward those with more severe manifestations compared with other published studies, which had higher proportions of asymptomatic infants (16, 22). A technical limitation was that T cell responses to viral antigens other than pp65 were not measured. Given the low number of PBMCs available from limited infant blood samples, pp65 was chosen as the target antigen pool due to its broad recognition by CD4^+^ and CD8^+^ T cells across most HLA haplotypes (45–49). It is also possible that other methods to identify antigen-specific T cells, such as ELISpots or tetramer staining, may have higher sensitivity than assays utilized in this study. In addition, the limited number of T cell markers evaluated for this study does not definitively prove the phenotypes of exhausted or naive T cells in this cohort, so these interpretations remain inferential. Another limitation is that the finding of changes in T cells is associated with progressive SNHL, but data that mechanistically connect it to this outcome are lacking. Finally, clinical outcomes were measured at 1–2 years of age, which might miss cases of loSNHL arising beyond this age.

In summary, this study shows that cCMV infection induces a range of T cell responses that lack antigen-specific cytokine-secreting function, some expressing markers that suggest T cell exhaustion and other lacking differentiation in response to in utero infection. Cytokine-secreting, CMV-specific CD4^+^ and CD8^+^ T cells arise during the second year of age in some children and are associated with decreased PD-1 expression. In this small cohort, a lack of in utero T cell differentiation was associated with later DD. Among a small group of infants, persistence of PD-1^+^CD8^+^ cells over time was observed among those with progressive SNHL. Further studies are needed to define the specificity of these T cells, to validate and extend these findings in larger cohorts of cCMV-infected children, and to investigate immune-mediated mechanisms by which cCMV infection affects long-term neurodevelopmental and hearing outcomes.

## Methods

### Sex as a biologic variable.

Infants of both sexes were included in both the control and infected populations. Study enrollment was not restricted based on sex.

### Patient population.

Patients were enrolled and samples were collected in this prospective case-control study from December 2019 to September 2022 at Nationwide Children’s Hospital (NCH). Infants with cCMV infection were identified by referral to the NCH Neonatal Infectious Disease Clinic due to clinical symptoms at birth, positive CMV testing after failed newborn hearing screening, or by positive saliva CMV PCR at admission screening in the NCH neonatal intensive care unit (NICU). All infants were diagnosed with cCMV infection by positive saliva or urine PCR before 3 weeks of age, which was confirmed by a second positive CMV PCR test (blood, urine, or dried blood spots obtained from the Ohio Department Health). Infants with CMV DNA detection beyond 3 weeks of age, or who had a positive first saliva PCR but negative second PCR of blood and/or urine and negative dried blood spot PCR were excluded from enrollment.

Infants with cCMV infection underwent clinical evaluation, including physical examination, laboratory work (complete blood count with differential and platelet count, ALT/AST, and total and direct bilirubin), head ultrasound and/or brain MRI. Infants were seen at 3-month intervals through the first year of age and annually thereafter. Infants were referred for early intervention services and underwent Bayley neurodevelopmental testing at 6- to 12-month intervals in the NCH Division of Neonatology High-Risk Infant Follow-up Clinic. Children were referred to the NCH Departments of Ophthalmology and Audiology for dilated eye examinations and serial follow-up hearing assessments every 6 months through age 3 years, with continued follow-up beyond 3 years if clinically indicated. SNHL was identified by auditory brain stem response testing in neonates and by age-appropriate behavioral and/or cochlear function testing in older children. Study data were collected and managed using REDCap electronic data capture tools hosted at Nationwide Children’s Hospital (https://projectredcap.org/). Serial blood samples were obtained at enrollment and at subsequent clinical visits.

### Study definitions.

Symptomatic cCMV infection at birth was defined by systemic findings identified on physical examination or laboratory testing (hepatosplenomegaly, petechial rash, intrauterine growth restriction, small for gestational age, chorioretinitis, SNHL, transaminitis, direct hyperbilirubinemia, thrombocytopenia), without or with CNS findings (microcephaly, periventricular calcifications, cortical dysplasia). Isolated eoSNHL was defined as SNHL identified through newborn hearing screening without other specific cCMV symptoms. Children without these findings were defined as having asymptomatic cCMV infection. DD was defined as Bayley III or IV testing below the average range in at least one domain or, for children for whom this formal testing was not available, clinical diagnosis by primary care provider or other subspecialty pediatrician. Language delays due to hearing loss were excluded from the definition of DD. SNHL was defined as an audiologic threshold above 25 decibels (dB) in either ear. Hearing thresholds of 26–40, 41–70, 71–90, and ≥91 dB were defined as mild, moderate, severe, and profound hearing loss. Progressive SNHL was defined as a hearing deterioration to a higher threshold in either ear, inclusive of loSNHL. The two outcomes, SNHL and DD, were defined at ≥12 months of age and were collectively termed as neurodevelopmental impairment.

### Control participants.

Uninfected infants were enrolled with parental informed consent in primary care clinics, the NICU, and in the NCH Infectious Disease clinic if evaluation for active infection was negative. Blood samples from anonymous adult volunteers were also collected and tested for CMV IgG by ELISA as described previously (50), and PBMCs of CMV-seropositive adults were included as a comparison group for CMV-specific T cell responses.

### Flow cytometry.

PBMCs were isolated from blood samples via Ficoll gradient (51) and stored in liquid nitrogen for batched analysis. For flow cytometry, thawed PBMCs were stained with fluorochrome-conjugated anti-human antibodies (Supplemental Table 2) purchased from Biolegend and BD Biosciences. Adult PBMCs were used to validate antibody staining in serial dilution in comparison with unstained controls, isotype control antibodies, and fluorescence-minus-one staining. For enrolled participants, aliquots of PBMCs were thawed and immediately stained for lineage markers (CD3, CD4, CD8, and CD16) and surface receptors (PD-1, CD57, CD28, and CD45RO), and expression frequencies were quantified. To assess CMV-specific T cell responses, additional aliquots of PBMCs were stained for T cell memory markers (CD45RA, CCR7) for 30 minutes at 37°C and then divided into 3 aliquots that were unstimulated or stimulated with either SEB (example data shown in Supplemental Figure 5) or a CMV pp65 peptide pool, which elicits both CD4^+^ and CD8^+^ T cell responses across most HLA haplotypes (JPT Peptide Technologies). PBMCs were incubated for 6 hours at 37°C with brefeldin A added after 1 hour, washed, stained for surface markers (CD3, CD4, CD8, and CD16) followed by permeabilization and staining for intracellular cytokines (TNF-α, IFN-γ, IL-2, and MIP-1β). Flow cytometry was performed using the Attune NxT cytometer with analysis and quantitation performed using FlowJo v10.8 Software (BD Life Sciences). Frequencies of CMV pp65-specific cytokine-secreting T cells were calculated by subtracting frequencies in unstimulated PBMCs from those of pp65-stimulated cells.

**Figure 6:**
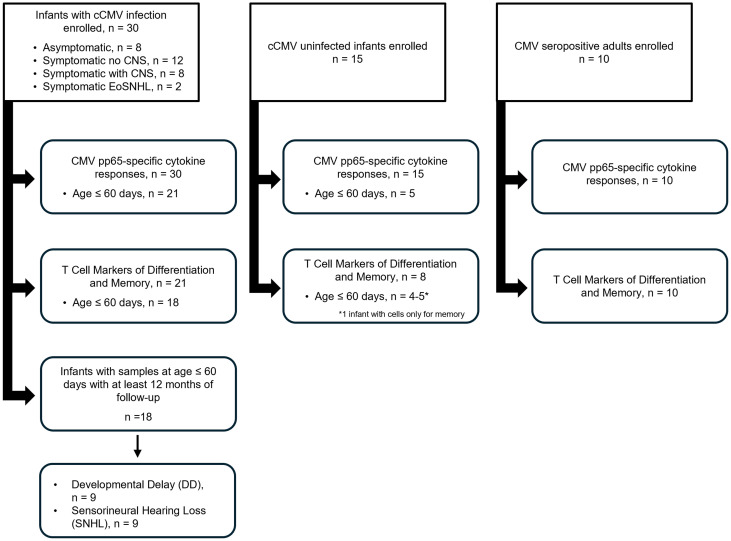
Consort diagram. Participant enrollment and experimental flow.

Memory subsets were defined as naive (CD45RA^+^CCR7^+^), Tcm (CD45RA^–^CCR7^+^), Tem (CD45RA^–^CCR7^–^), or Temra (CD45RA^+^/CCR7^–^) CD4^+^ or CD8^+^ T cells. For surface staining with PD-1, CD45RO was used as a marker of memory cells in lieu of CD45RA/CCR7 owing to differences in the CCR7 staining requirements. A consort diagram of participant enrollment and experimental flow is provided in Figure 6.

Flow cytometry data were primarily displayed as frequencies of CD4^+^ or CD8^+^ T cells expressing markers of interest as a percentage of the appropriate cell population due to the known variation of white blood cell count by age given the longitudinal nature of the project and incorporation of adult comparative data. To demonstrate equivalency of cell frequencies between participants, corresponding absolute white blood cell and lymphocyte counts were obtained using a hematology analyzer (Sysmex Inc.) and are shown in Supplemental Figures 2, 4, and 6.

### CMV viral load quantitation.

Genomic DNA was isolated from plasma using the QIAmp DNA Blood Mini Kit (Qiagen Sciences). Quantitative DNA PCR was performed using primers and probes for HCMV immediate-early 2 (IE2) exon 5 in comparison with a standard curve generated by a plasmid encoding the IE2 exon 5 sequence (Invitrogen Life Technologies) (52) using a StepOnePlus Real-Time PCR system (Applied Biosystems) (53). Viral loads were expressed as copies/mL of plasma.

### Statistics.

Comparative and descriptive statistics were performed using GraphPad Prism v8.0.0 for Windows. Categorical variables were compared using the Fisher’s exact test while continuous variables were compared using the Mann Whitney *U* test, the Kruskal-Wallis test with Dunn’s comparisons for pairwise analysis, or 2-way ANOVA as appropriate. Data were graphed displaying median ± 95% CI. Longitudinal data were analyzed using linear mixed models generated in R (R Core Team, https://www.R-project.org/) using package *nlme* (54, 55). Group and subgroup comparisons for visualization were conducted in Python using numpy for descriptive statistics. Subgroups were plotted as convex hulls of the subgroup data and non-DD versus DD groups plotted as the true ellipsoidal variant of the standard deviational ellipse at 1 SD (56, 57). Polyfunctionality of the T cells was analyzed using SPICE software version 6.1 (https://niaid.github.io/spice/) (58) and calculated as a PI as described previously (59). *P* values of less than 0.05 were considered significant.

### Study approval.

This prospective observational study was approved by the NCH Institutional Review Board (NCH IRB no. 00000701). All infants were enrolled after parental informed consent.

### Data availability.

Values for all data points in graphs are reported in the Supporting Data Values file.

## Author contributions

AKM designed the studies, conducted the experiments, acquired and analyzed the data, and drafted the manuscript. RD designed the studies, conducted the experiments, acquired and analyzed the data, and reviewed and revised the manuscript. PJS acquired the data and reviewed and revised the manuscript. KF and BG conducted the experiments, acquired and analyzed the data, and reviewed and revised the manuscript. TP, RC, and WCR acquired the data and reviewed and revised the manuscript. CCP and JRH designed the studies and reviewed and revised the manuscript. JP analyzed the data and reviewed and revised the manuscript. UF, PM, and OA acquired the data and reviewed and revised the manuscript. MS designed the studies, analyzed the data, and reviewed and revised the manuscript.

## Supplementary Material

Supplemental data

ICMJE disclosure forms

Supporting data values

## Figures and Tables

**Table 1:**
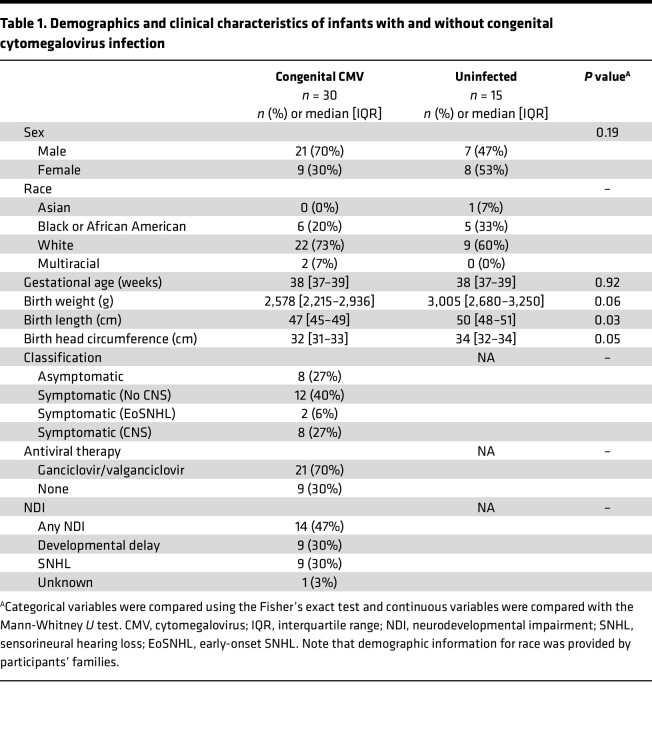
Demographics and clinical characteristics of infants with and without congenital cytomegalovirus infection

## Version 1 09/24/2024

Electronic publication
