# Supplementary material for: T cell responses and clinical symptoms among infants with congenital cytomegalovirus infection
Source: JCI Insight. 2024 Sep 24;9(18):e171029. doi: 10.1172/jci.insight.171029 (PMC11457853; doi:10.1172/jci.insight.171029)
Supplement: Supplemental data [file jciinsight-9-171029-s237.pdf]

Supplementary Information

Supplementary Tables

Table S1. Summary of blood samples collected according to age.

Age (months)	ALL	0-2	3-8	9+
CMV (n)	30	21	18	9
Control (n)	15	7	3	5

Table S2. Flow cytometry panels and fluorochrome-conjugated antibodies.

Laser	Marker	Fluorophore	Clone ID	Company	Catalog #
Surface Repertoire Panel					
405	Anti-CD8a	BV510	RPA-T8	Biolegend	300460
405	Anti-CD3	BV605	UCHT1	Biolegend	303528
405	Anti-CD57	BV785	QA17A04	Biolegend	302906
488	Anti-CD28	FITC	CD28.2	Biolegend	621614
488	Anti-PD-1	PerCP/Cy5.5	A17188B	Biolegend	359412
561	Anti-CD45RO	PE/Dazzle 594	UCHL1	Biolegend	317414
561	Anti-CD4	PE/Cy7	OKT4	Biolegend	302610
640	Anti-CD16	APC-Cy7	3G8	BD Bioscience	557758
640	Fixable Viability Stain 780	N/A	N/A	BD Bioscience	565388
Intracellular Cytokine Staining (ICS) Panel					
405	Anti-IFN-γ	BV421	4S.B3	Biolegend	301048
405	Anti-CD8a	BV510	RPA-T8	Biolegend	300460
405	Anti-CD3	BV605	UCHT1	Biolegend	328640
405	Anti-CD107a (LAMP-1)	BV711	H4A3	Biolegend	353230
405	Anti-CD197 (CCR7)	BV785	G043H7	Biolegend	304106
488	Anti-CD45RA	FITC	HI100	Biolegend	621614
488	Anti-CD279(PD-1)	PerCP/Cy5.5	A17188B	Biolegend	550078
561	Anti-MIP1β (CCL4)	PE	D21-1351	BD Bioscience	304248
561	Anti-CD45RO	PE/Dazzle 594	UCHL1	Biolegend	317414
561	Anti-CD4	PE/Cy7	OKT4	Biolegend	500310
640	Anti-IL-2	APC	MQ1-17H12	Biolegend	502928
640	Anti-TNF-α	AF700	MAb11	Biolegend	565388
640	Fixable Viability Stain 780	N/A	N/A	BD Bioscience	565388

A.

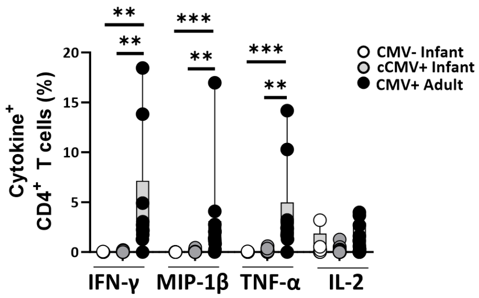

B.

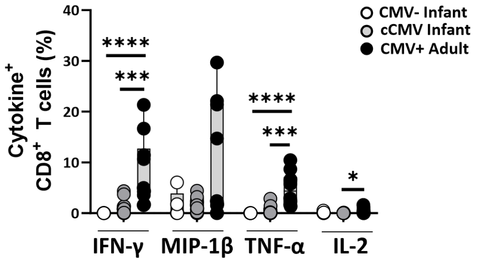

Figure S1. T cell responses to SEB in cCMV infected neonates, uninfected infants, and CMV seropositive adults. Peripheral blood mononuclear cells (PBMCs) of cCMV infected infants (n = 21) or uninfected infants (n = 5) at age ≤ 60 days and CMV seropositive adults (n = 10) were unstimulated or stimulated with staphylococcal enterotoxin B (SEB) analyzed by intracellular cytokine staining and flow cytometry. Frequencies of cytokine secreting cells in unstimulated conditions ("background") were subtracted from those after SEB stimulation. The background-subtracted frequencies of cytokine-secreting cells after SEB stimulation are shown as frequencies of $CD4^+$ (**A**) and $CD8^+$ (**B**) T cells expressing IFN-γ, MIP-1β, TNF-α, and IL-2 for CMV uninfected infants (CMV- infant), cCMV infected infants (CMV+ infant), and CMV seropositive adults (CMV+ adult). Groups were compared using the Kruskal-Wallis test with Dunn's multiple comparisons for all panels. * $p < 0.05$; ** $p < 0.01$; ns, not significant ($p > 0.05$).

A.

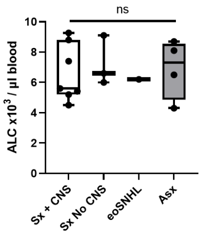

	Asx	eoSNHL	Sx No CNS	Sx With CNS
N	4	1	3	7
ALC x 10^3/µl, median [IQR]	6.5 [4.3-8.7]	6.2 [n/a]	6.6 [6.0-9.1]	5.6 [5.2-8.8]

B.

C.

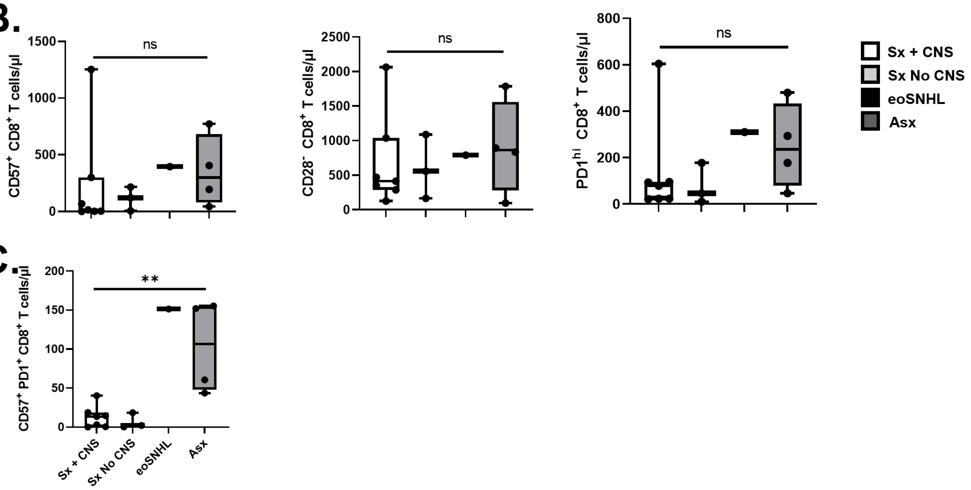

Figure S2. Cell quantities of CD8$^+$ T cells expressing markers of differentiation in cCMV infected infants according to clinical symptoms at birth. The cCMV infected infants were classified according to clinical symptoms as Symptomatic with CNS findings (Sx + CNS, n=7), Symptomatic without CNS findings (Sx No CNS, n=3); isolated early onset SNHL (eoSNHL, n=1), or asymptomatic (Asx, n=4) at birth. PBMCs of at age ≤ 60 days were analyzed by flow cytometry for frequencies of global populations of CD8$^+$ T cells expressing CD57, CD28, PD-1. (A) Absolute lymphocyte counts (x 10^3/µl blood) were derived from the complete blood counts and compared between groups. (B) Absolute quantities of C57$^+$, CD28$^-$, or PD-1$^+$ CD8$^+$ T cells per µl blood were calculated using flow cytometry frequencies of CD3$^+$ CD8$^+$ T cells expressing the marker of interest and compared between clinical symptom groups. (C) Absolute quantities of C57$^+$ PD-1$^+$ CD8$^+$ T cells were calculated and compared between groups. Groups were compared using the Kruskal-Wallis test with Dunn's multiple comparisons for all panels. * $p < 0.05$; ** $p < 0.01$; ns, not significant ($p > 0.05$).

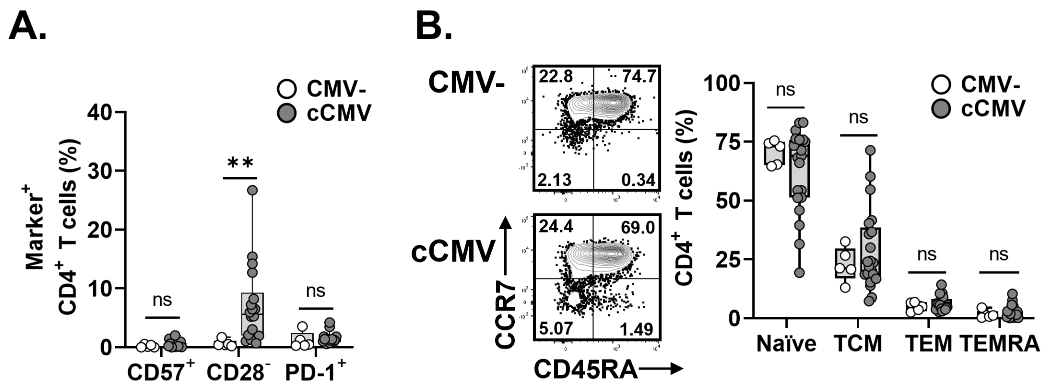

Figure S3. Global CD4⁺ T cell differentiation and memory in cCMV infected infants. PBMCs from infants at age ≤ 60 days were analyzed by flow cytometry for (**A**) frequencies of $CD57^+$, $CD28^-$, or $PD\text{-}1^+$ $CD4^+$ T cells (cCMV infected, n = 18; uninfected, n = 4), or (**B**) CCR7/CD45RA memory subsets (cCMV infected, n = 21; uninfected, n = 5). Groups were compared using the Mann-Whitney U test for all panels. * p<0.05; *** p<0.001; ns, not significant (p > 0.05).

A.

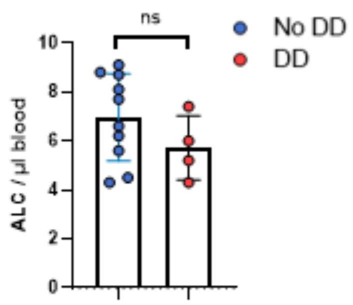

C.

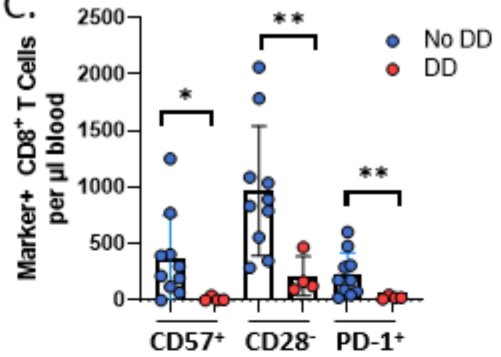

B.

	No DD	DD
N	10	4
ALC x 10^3/µl, median [IQR]	7.15 [5.325-8.725]	5.6 [4.525-7.05]

Figure S4. Cell quantities of CD8$^+$ T cells expressing markers of differentiation in cCMV infected infants according to developmental outcome. Of the cCMV infected infants with or without developmental delay who had CBCs performed (DD, n=4; No DD, n=10), groups were compared for (A-B) absolute lymphocyte counts (x 10^3/µl blood), and (C) absolute quantities of C57$^+$, CD28$^-$, or PD-1$^+$ CD8$^+$ T cells per µl blood. Groups were compared using the Mann-Whitney U test for all panels. * $p < 0.05$; ** $p < 0.01$; ns, not significant ($p > 0.05$).

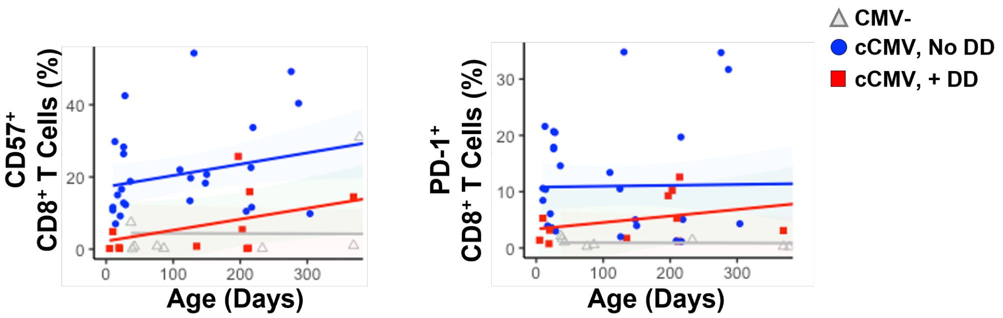

Figure S5. Longitudinal comparison of CD8$^+$ T cells according to developmental outcome.

For all infants with longitudinal samples available for analysis, frequencies of CD57$^+$ or PD-1$^+$ CD8$^+$ T cells are shown over the first 365 days of age for those with no DD (n = 14), or DD (n = 4) with 95% confidence intervals shown as shaded areas using linear mixed models.

A.

	No Hearing Loss	Nonprogressive hearing loss	Nonprogressive hearing loss
N	12	5	3
ALC x 10^3/µl, median [IQR] (min-max)	7.2 [5.4-9.1] (2.3-10.96)	7.55 [5.1-8.1] (3.9-9.9)	7.2 [5.5-9.2] (3.7-10.4)

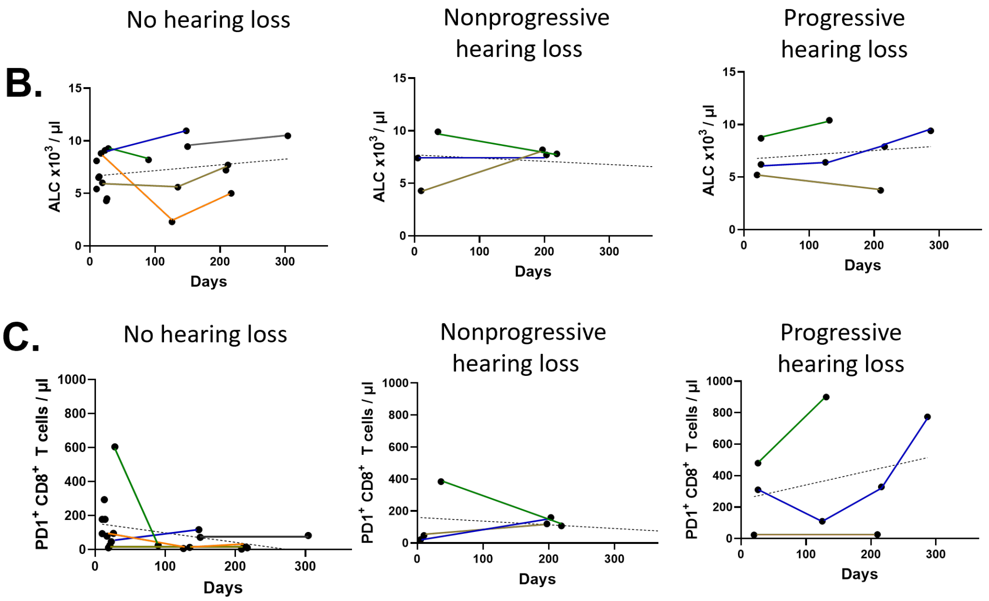

Figure S6. Longitudinal Absolute lymphocyte counts and PD-1$^+$ CD8$^+$ T cells according to hearing outcome. For cCMV infected infants with available CBCs, those with no hearing loss (n=12) were compared to those with nonprogressive (n=5) or progressive SNHL (n=3). (**A-B**) Absolute lymphocyte counts (x 10^3/µl blood) are shown for infants with no hearing loss, nonprogressive SNHL, and progressive SNHL. In panel B, repeated observations over time are linked with colored lines representing individual subjects. (C) Absolute quantities of PD-1$^+$ CD8$^+$ T cells per µl blood were calculated based on flow cytometry frequencies of CD3$^+$ CD8$^+$ T cells expressing PD-1. Repeated observations over time are connected with colored lines corresponding to the same subjects shown within each group in panel B. Linear regression shown as dotted lines.

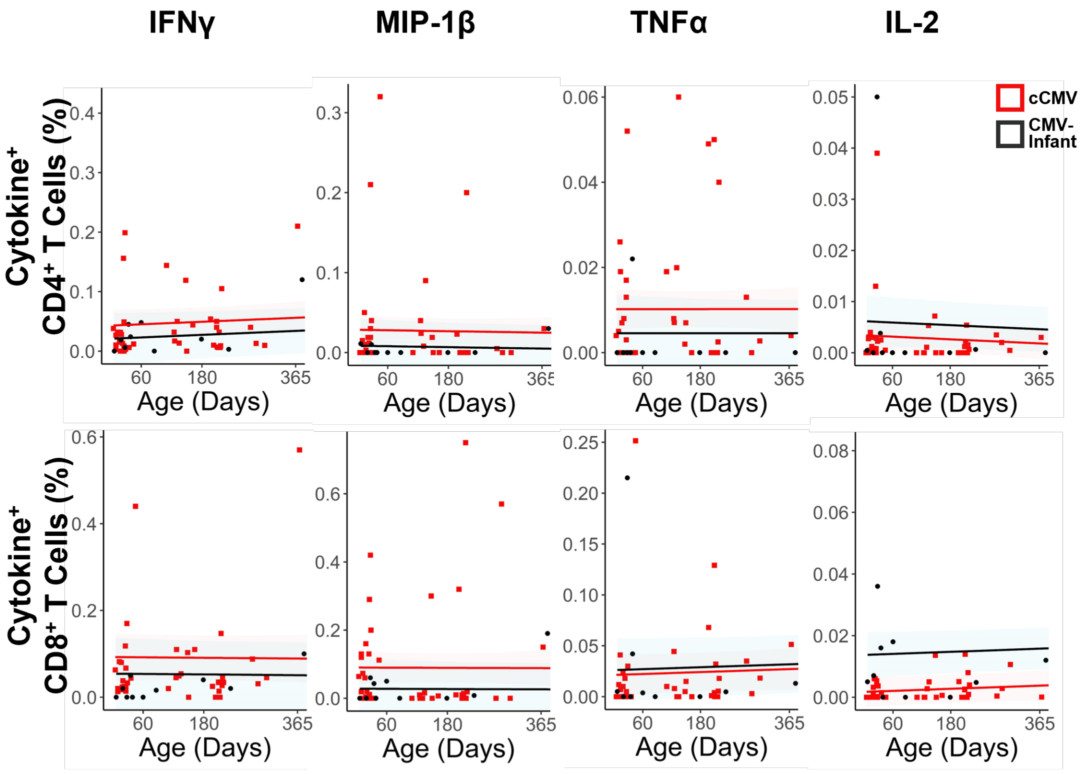

Figure S7. Frequencies of CMV pp65-specific cytokine expressing CD4^{+} and CD8^{+} T cells over the first year of age. Frequencies of CD4^{+} and CD8^{+} T cells expressing IFN-γ, MIP-1β, TNF-α, or IL-2 after pp65 stimulation are shown for CMV uninfected infants (black, n=30) or cCMV infected infants (red, n=13) over 365 days. 95% confidence intervals shown as shaded areas using linear mixed models.

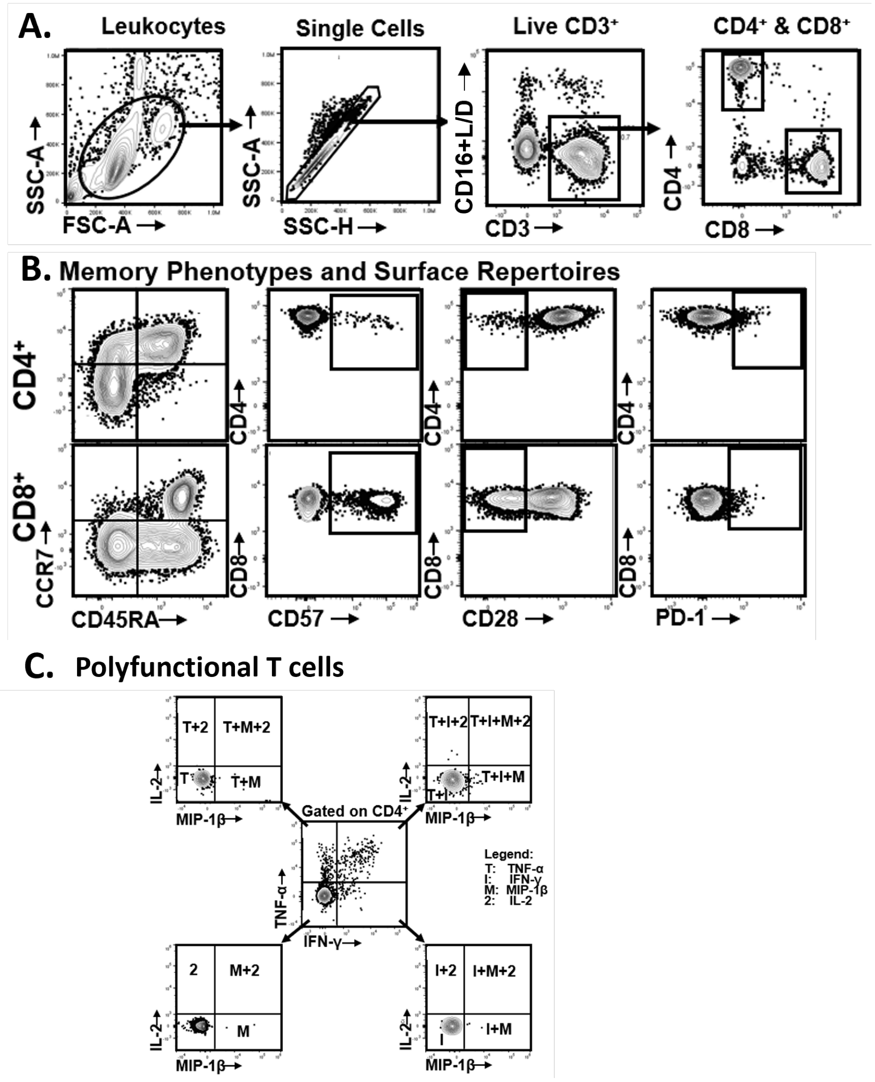

Figure S8. Gating Strategy

Debris and doublets were excluded using forward and side scatter area vs. height. Singlets were gated for viable CD3$^+$ T cells, followed by gating for CD4$^+$ and CD8$^+$ cells. Within the CD4$^+$ or CD8$^+$ population, CD45RA and CCR7 were used to define naïve (CD45RA$^+$CCR7$^+$), central memory (T_{CM}; CD45RA$^-$CCR7$^+$), effector memory (T_{EM}; CCR7$^-$CD45RA$^-$) and TEMRA (CCR$^-$ CD45RA$^+$) populations. Independently, CD4$^+$ or CD8$^+$ T cells were gated for CD57$^+$, CD28$^-$, or PD-1$^+$ cells. To determine polyfunctionality, CD4$^+$ T- cells were first gated for TNF-α and/or IFN-γ expression. Each quadrant of TNF-α/IFN-γ gating were further gated for IL-2 and/or MIP-1β. The frequencies of single vs. multiple cytokine expressing cells were expressed as percentages of total CD4$^+$ T cells. For SPICE analysis, frequencies of single and multiple cytokine expressing CD4$^+$ T cells were independently obtained using Boolean gating within FlowJo^{TM} software.
